# A Coarse-to-Fine Framework for Multiple Pedestrian Crossing Detection

**DOI:** 10.3390/s20154144

**Published:** 2020-07-25

**Authors:** Yuhua Fan, Zhonggui Sun, Guoying Zhao

**Affiliations:** 1 School of Mathematical Science, Liaocheng University, Liaocheng 252000, China; sunzhonggui@lcu.edu.cn; 2 Center for Machine Vision and Signal Analysis, University of Oulu, University of Oulu, 90570 Oulu, Finland; guoying.zhao@oulu.fi

**Keywords:** pedestrian crossing, detection, probe vehicle video, coarse-to-fine

## Abstract

When providing route guidance to pedestrians, one of the major safety considerations is to ensure that streets are crossed at places with pedestrian crossings. As a result, map service providers are keen to gather the location information about pedestrian crossings in the road network. Most, if not all, literature in this field focuses on detecting the pedestrian crossing immediately in front of the camera, while leaving the other pedestrian crossings in the same image undetected. This causes an under-utilization of the information in the video images, because not all pedestrian crossings captured by the camera are detected. In this research, we propose a coarse-to-fine framework to detect pedestrian crossings from probe vehicle videos, which can then be combined with the GPS traces of the corresponding vehicles to determine the exact locations of pedestrian crossings. At the coarse stage of our approach, we identify vanishing points and straight lines associated with the stripes of pedestrian crossings, and partition the edges to obtain rough candidate regions of interest (ROIs). At the fine stage, we determine whether these candidate ROIs are indeed pedestrian crossings by exploring their prior constraint information. Field experiments in Beijing and Shanghai cities show that the proposed approach can produce satisfactory results under a wide variety of situations.

## 1. Introduction

With the advent of smart phones and location-based services, there has been an increased interest in providing route guidance to pedestrians under different environments [1,2,3]. As an important part of transportation infrastructures, pedestrian crossing serves to secure pedestrians’ lives and possessions and keep traffic flow in order [4]. What’s more, in the generation of walking paths, one of the major safety considerations is to ensure that streets are crossed at places with established pedestrian crossings. Therefore, map service providers are keen to know the exact locations of pedestrian crossings in the city. With the proliferation of probe vehicle usage, researchers have started to develop models that can detect pedestrian crossings from probe vehicle videos. Once a pedestrian crossing is detected, its location can be obtained from the corresponding GPS trace logged simultaneously with the video.

The detection of pedestrian crossings from probe vehicles typically rely on vision interesting feature cues such as geometric characteristics, and color variation information, which capture the distribution patterns of pedestrian crossings in traffic scenes. For example, Se [5,6] detects pedestrian crossings using lines grouping characteristics and projective geometry constraints. Boudet and Midenet [7] use videos to detect pedestrian crossing patterns through the fusion of sensor data. Liu et al. [4,8] use videos to automatically detect pedestrian crossing and analyze its impairment status based on classifiers. A thorough literature review is provided in Section 2.

**Figure 1:**
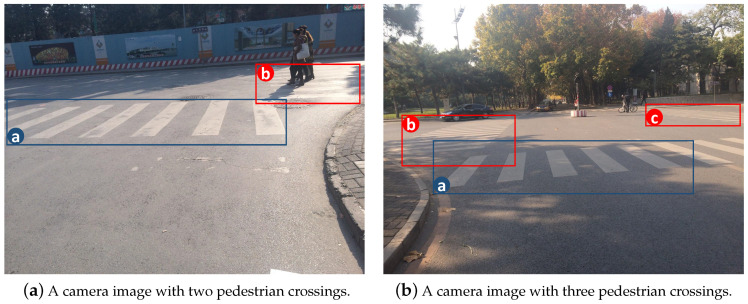
Existing methods focus on detecting the pedestrian crossing immediately in front of the camera (indicated by the blue rectangle), while leaving the other undetected (indicated by the red rectangles).

Most, if not all, literature in this field focuses on detecting pedestrian crossings immediately in front of the camera, which causes an under utilization of the video information. For example, although there are two pedestrian crossings in Figure 1a, existing literature would only try to detect the one inside the blue rectangle, while leaving the one in the red rectangle undetected. Similarly, in Figure 1b, only the pedestrian crossing in the blue rectangle will be detected despite of the fact that there are actually three in this camera image. The situations illustrated in Figure 1a,b are not uncommon in many cities. Based on 10 probe vehicle videos collected in Beijing and Shanghai, we find that about 33% of the intersections have more than one pedestrian crossings.

In this research, we propose a coarse-to-fine framework that detects all pedestrian crossings in a camera image. This means that the two pedestrian crossings in Figure 1a and all three pedestrian crossings in Figure 1b should be detected. This allows us to extract all pedestrian crossing information from the videos. Since more than one pedestrian crossings need to be detected, at the coarse stage, we search for multiple vanishing points, each of which corresponds to one pedestrian crossing [9], to obtain candidate regions of interests (ROIs). Later, at the fine stage, we determine whether these candidate ROIs are indeed pedestrian crossings by exploring their prior constraint information. We validate the detection algorithm using 10 videos recorded by probe vehicles in Beijing and Shanghai. These videos are first converted to camera images by retaining one frame every five frames. We then train our model and carry out extensive experiments on these images under different traffic and weather conditions. We show that satisfactory results can be obtained under our approach.

The remainder of this paper is organized as follows. In Section 2, we review related literature. In Section 3, we present the coarse-to-fine framework for the detection of multiple pedestrian crossings. In Section 4, we validate our approach using videos recorded by probe vehicles in Beijing and Shanghai. Finally, we conclude our work in Section 5.

## 2. Related Work

As an important subsystem in intelligent transportation systems, pedestrian crossing detection based on computer vision has become an important research area. Franke and Heinrich [10] considered pedestrian crossing detection as an important goal of vision-based technologies to reduce traffic dangers. Compared to landmark-based pedestrian navigation [11], pedestrian crossings based navigation is particularly useful for individuals who are partially sighted when it is integrated with a standard map-based system.

Pedestrian crossings take different forms in different countries. In most countries, pedestrian crossings are painted as evenly-spaced white stripes that are parallel to each other. Their most prominent characteristics are edge and lines feature information. Leveraging this information, Uddin and Shioyama [12] and Choi et al. [13] utilized projective geometry constraints to design the detection algorithms. Unfortunately, the bipolarity feature they used lacks stability for different pedestrian crossings and the method does not adapt well to different road environments. Coughlan and Shen [14] proposed a method for grouping geometric features to detect pedestrian crossings, and tested their algorithms using a database of images photographed by an unguided blind pedestrian. This approach performs poorly when confronted with multiple pedestrian crossings in real-world traffic settings. Although Se [5] has fully considered the differences in pedestrian crossings and stairs in the use of crosswalk detection as a tool for outdoor navigation in mobility aids for the partially sighted, it could be of value for using in pedestrian crossing detection.

Fang et al. [15] and McCall [16] used the temporal and spatial information from video sequences to work toward a vehicle safety system in complex driving environments. Salmane et al. [17] explored a video-based railway-road safety system for detecting hazard situations at level crossings. Additionally, Boudet and Midenet [7] performed spatial measurements on areas along a pedestrian crossing using traffic-oriented video sensors, supporting the occupancy patterns to exploit redundant information conveniently for pedestrian crossing detection; they used an evidence-based data fusion process to obtain redundant information by combining intra-sensor or inter-sensor data. Chyi-Ren et al. [18] used environmental feature vectors to detect only one crossing in an image and determine the crossing area based on the environmental parameters. This method can detect pedestrian crossing very well because of the high quality of images.

Although significant efforts have been made to implement pedestrian crossing detection, approaches in the literature focuses on detecting crosswalks immediately in front of the camera, while causing an under utilization of the video information. In this research, we propose a coarse-to-fine framework that detects all pedestrian crossings in a camera image. Extensive experiments are also carried out to have a comparison to other prior methods to show the superior performance of our method under the same experiment condition.

## 3. Proposed Approach

**Figure 2:**
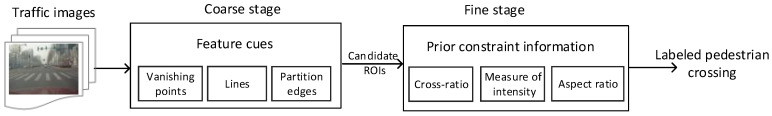
Operation flowchart, showing the coarse and fine stages of the proposed pedestrian crossing detection method.

The primary goal of our approach is to detect multiple pedestrian crossings that appear in a camera captured image in a robust and efficient way. Traffic scenes often contain complex semantic details such as pedestrians, vehicles and trees. These distractions make pedestrian crossing detection a difficult task. We develop a coarse-to-fine framework, which considers visual cues and a priori constraint information of a pedestrian crossing simultaneously, while preserves the discriminative detection information. At the coarse stage, coarse detection and rough localization are performed, including searching for vanishing points, detecting straight lines, and partitioning edges to obtain rough candidates for the region of interest. Later, at the fine stage, spatiotemporal information refinement and fine localization are performed, including identification of the geometric and statistical constraints of the pedestrian crossings. Figure 2 shows the flowchart of presented method for multiple pedestrian crossing detection.

### 3.1. Coarse Stage

At the coarse stage, we address two key issues: the rapid estimation of vanishing points for pedestrian crossings and the robust extraction of line features, which allows us to partition edges and avoid unnecessary search for crosswalks.

#### 3.1.1. Searching for Vanishing Points

**Figure 3:**
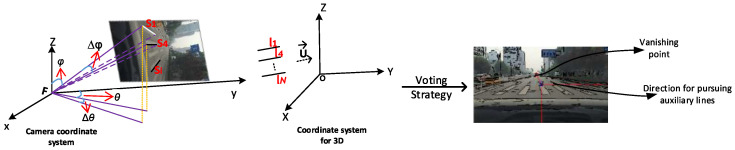
Searching for vanishing points and their directions in a traffic scene.

Vanishing point detection is an important technique in the fields such as road detection, camera calibration and visual navigation [19,20,21,22,23]. Vanishing point be estimated using the symmetrical features of the surrounding structure in the image [19]. Two methods are commonly used to search the vanishing points: The first one searches for vanishing points based on Hough transform [19,20,24]. In this approach, after obtaining line features using Hough transform, the vanishing point will be the point at which the majority of the supporting line segment primitives intersect. The second method considers vanishing point computation as a statistical estimation problem [9,21,22,25,26]. In most cities, a pedestrian crossing is characterized by a set of parallel stripes. When captured on a camera image in the vehicle mode, the pedestrian crossing will show some geometric deformation due to the imaging mechanism of camera lens. This will make the extensions of the stripes converge to a vanishing point [21]. Although the accuracy of the above methods for vanishing point detection can be guaranteed, it is expensive in terms of computational costs during the voting stage because each pixel can be regarded as both a voter and a vanishing point candidate. Most of all, these methods can not search for multiple vanishing points efficiently in the image plane. Therefore, these methods cannot meet the requirements of multiple pedestrian crossings detection. Our approach is very efficient with low computational complexity and high accuracy for detecting multiple vanishing points in the structured road images. Figure 3 illustrates the search process.

When searching for vanishing points, we take different situations into account to provide supporting knowledge for pedestrian crossing detection. With too many parallel lines, identifying the desired points from the false ones can be difficult. Based on the conventional Hough transform, every line intersection on the image plane that corresponds to a circle intersection on the Gaussian sphere [27] can represent a 3D direction of the traffic scene, or one vanishing point. Thus, when searching for vanishing points, instead of looking for the convergence of lines on the image, we can look for the intersections of circles on the Gaussian sphere associated with the image.

For Figure 3, there are a total of *N* segments detected, which converge to the vanishing point associated with $\vec{U}$. The supporting parallel lines $l_{1},l_{2},\cdots ,l_{N}$ in the 3D space have the same structural and geometric distribution. Meanwhile, assume that the coordinate frame is associated with 3D scene and camera by (O,X,Y,Z) and (F,x,y,z), respectively. We can denote θ and φ as the angles formed by segment and camera coordinate system, respectively. The Gaussian sphere can be defined as a great circle representing the interpretation plane of supporting segments.

According to the structural character of the pedestrian crossing, We assume that at least k(k≥N/2) among these *N* lines would meet at vanishing points $V_{j},j\geq 0$. For the lines that support segment $S_{i}$, a great circle can be drawn on the Gaussian sphere Γ, centered at the optical center of the camera. This circle represents all the 3D directions $\vec{U}$ that projects on the image line; the trace of the corresponding segment on the Γ in the direction $\vec{U}$ that can be projected on $S_{i}$. For the given vector ${\vec{U}}_{i}\left(\theta_{i},\varphi_{i}\right)$, all the possible vectors $\vec{N}\left(\theta_{u},\varphi_{u}\right)$ satisfy the condition on Γ: $\cos\left(\theta_{i}-\theta_{u}\right)\sin\varphi_{i}\sin\varphi_{u}+\cos\varphi_{i}\cos\varphi_{u}=0,\left(\theta_{u}\in \left[0,2\pi \right),\varphi_{u}\in \left[0,\pi /2\right)\right)$ [28]. Each line intersection on the image plane corresponds one circle intersection on the Gaussian sphere, which represents one vanishing point. With this method, we avoid the problem of finding an infinite number of vanishing points.

For the geometrical of the pedestrian crossing, we utilize an equal-size-cell quantization of Hough space, without providing many cells of equal surfaces on Γ. This method is applied to quantize the Hough space by using a regular quantization on θ and φ. On the Gaussian sphere, a cell of size Δθ and Δφ has a surface of ΔS=Δθ[cosφ−cos(φ+Δφ)]. Therefore, if the number of directions $\vec{U}$ is infinite, a uniform count on each cell will be obtained successfully.

**Figure 4:**
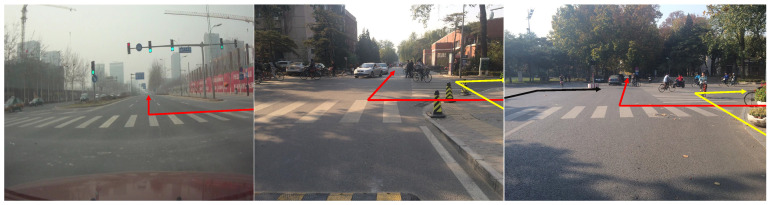
Presentations of pedestrian crossings in real traffic settings and their vanishing points (the location of arrow). (From left to right are the one, two, three pedestrian crossings in different traffic images and their corresponding vanishing points respectively.)

After the previous steps, we have a list of directions ${\vec{U}}_{k}$, each of which corresponds to a possible vanishing point in the image plane and is ranked by their counts in the accumulator space. According to the previous analysis, we do not need to consider the directions of multiple pedestrian crossings. By the counts in the accumulator space and ranking, this methodology can track all existing vanishing points in camera images. Figure 4 shows the vanishing points and their corresponding direction for pursuing auxiliary lines for presentations of several pedestrian crossings. Different locations and orientation of arrows searched three vanishing points for multiple pedestrian crossings. Note that the red, yellow, and black arrows are the searched three vanishing points.

#### 3.1.2. Line Extraction

Hough [29] presented Hough transform (HT) as an efficient tool for detecting lines, exploiting the point-line duality to identify the supporting lines of sets of collinear pixels in images. Since a pedestrian crossing in street can be viewed as an event that occupies state patterns on a region of interest and lasts for several seconds. However, in real-world traffic scenes, not all pedestrian crossings are occupied, and not all will have local or global pattern characteristics. For these, lines extraction will be extremely useful for multiple pedestrian crossing detection. Se [5] utilized this method to detect lines feature to obtain supporting information for the detection of pedestrian crossings. Liu [8] proposed a generalized Hough transform that treats a Radon transform as a linear transform first, and then facilitates parallel implementation of the Radon transform for multiple images. We can empirically find that the classical Hough transform method detects the lines more slowly and is easily interfered with by the noise in real road image. And another serious drawback is that the traditional Hough approach will guide a lot of error detection result in the objective areas with high edge density, such as in regions where there are lots of fences or buildings. Here, we consider the detection of edge points and the alternating pattern of pedestrian crossing to employ a more efficient improved Hough method.

By mapping each feature pixel to a set of lines potentially passing through the given pixel in the parameter space, the problem of identifying line patterns is converted into the simpler problem of seeking peaks in a vote map. In the traditional Hough transform method, a straight line is specified in Hough space as a pari (d,θ): this defines the line made up of all points (u,v) such that n(θ)·(u,v)=d, where n(θ)=(cos(θ),sin(θ)) is the unit normal vector to the line. Based on the Canny operator, we can find all edge points in the image. And then quantize the parameter space $\left(m,c\right)$ into a two-dimensional matrix H with appropriate quantization levels. When increment each element of list by 1, each element of $H\left(m_{i},c_{i}\right)$ can be correspond to an edge point. The result is a vote matrix showing the frequency of edge points corresponding to certain $\left(m,c\right)$ values.

In real traffic scenes, pedestrian crossings are always the regions that appear with groups of lines. At the same time, these lines may be disturbed by neighbors, partial occlusion, deformation, and so on. Thus, traditional line detection is unreliable as a pre-processing step, especially in cases where there are noisy or missing data in the traffic images. Due to inaccuracies in the voting scheme of the traditional Hough transform, we will taking into account these errors and generalize the Hough transform by using a new voting scheme as in [30]. According to the position character of pedestrian crossing in traffic images, we consider the lower two-thirds of the image, and this will accelerate the computation.

For lines extraction in the alternating pattern of black and white stripes, edges can be partitioned into two sets of equally spaced parallel lines according to the variation of their intensities. Because some invalid line segments should be removed and do not participate in the voting, this strong constraint for structures of pedestrian crossings can be used for eliminating the false ones [5]. By checking the changes of intensity from white to black (labeled white) and from black to white (labeled black), we can refine the lines to identify the potential structure of the pedestrian crossing and obtain the initial candidate ROIs. For the disturbances of noise and lens imperfections, the perspective projection of line segments are not congruent with the line segments detected in the image, thus the auxiliary lines along to the vertical direction will be helpful for confirming the existence of a pedestrian crossing.

**Figure 5:**
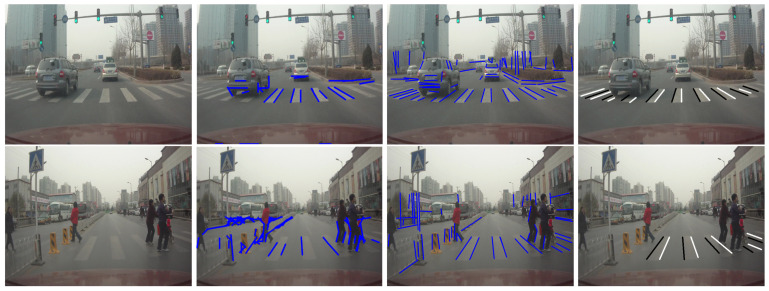
Examples of line feature detection results using traditional HT, improved HT, and edge partition based on improved HT. (First column: original image; Second column: line extraction results using tradition HT; Third column: line feature detection using improved HT; Forth column: edge partition results based on improved HT.)

Figure 5 shows examples of line feature extraction using different Hough transform and the edge partition results from the improved HT. The second column and the third column are the detection result of traditional HT and the improved HT respectively. We can empirically find that the improved HT can detect lines effectively and avoid produce spurious lines from pedestrian crossing effectively. For the traditional HT, clutter lines from noises tend to overlook some important objective lines from crosswalks.

### 3.2. Fine Stage

At the fine stage, we verify and refine candidate ROIs to obtain the true patterns of pedestrian crossings. For the disturbances of lane lines, handrails or emergency parking area, Utcke [31] pointed out that priori constraints can be incorporated to speed up processing by removing such extraneous information. In this section, we explore three prior constraints to eliminate false alarms without accidentally discarding important pedestrian crossing cues.

#### 3.2.1. Cross-Ratio Constraint

The cross ratio forms the basis of an ordinary system for recognizing quadruples of collinear image points. The performance of the system depends on the choice of rules for deciding whether four image points have a given cross ratio $C_{r}$. For pedestrian crossings, the cross ratio is a useful fundamental characteristic that remains unchanged under projection to an image; this is an effective and fundamental visually based numerical property for pedestrian crossing detection.

**Figure 6:**
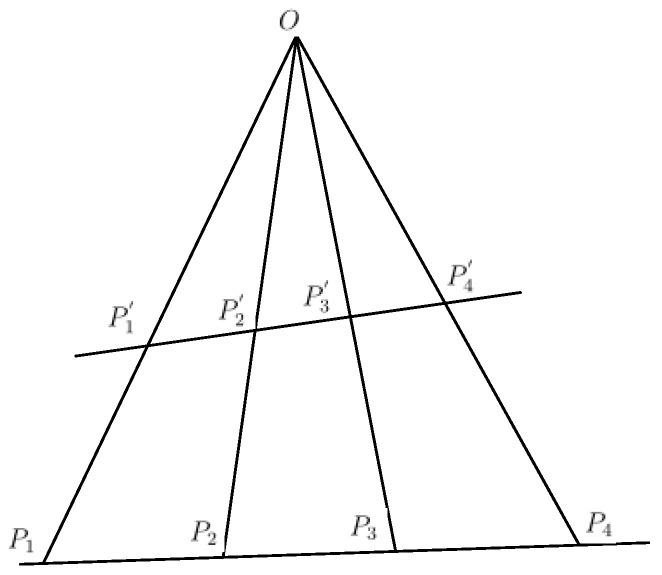
Illustration of cross-ratio constraint in the image plane.

Figure 6 illustrates the basic concept of a cross-ratio. Given four collinear points $P_{1}$, $P_{2}$, $P_{3}$ and $P_{4}$ in image space $P^{2}$, denote the Euclidean distance between two points $P_{i}$ and $P_{j}$ as $\Delta_{P_{i}P_{j}}$. Then, the cross ratio with respect to the vanishing point *O* is defined as follows:

(1) $$\begin{array}{c} C_{r}\left(P_{1},P_{2};P_{3},P_{4}\right)=\frac{\Delta_{P_{1}P_{2}}\Delta_{P_{3}P_{4}}}{\Delta_{P_{1}P_{3}}\Delta_{P_{2}P_{4}}} \end{array}$$

The four corresponding image points $P_{1}^{{}^{\prime }}$, $P_{2}^{{}^{\prime }}$, $P_{3}^{{}^{\prime }}$ and $P_{4}^{{}^{\prime }}$ for the points $P_{1}$, $P_{2}$, $P_{3}$, $P_{4}$ in the space will remain the cross-ratio invariable. For the pattern of a pedestrian crossing, we will consider the segments that are the edges of white bands and the black bands. In terms of projective invariant for four consecutive points, a relatively stable ratio can be found from equation 1.

We find that groups of lines in pedestrian crossings are often disturbed by stains, partial occlusion, or changes in illumination. After extracting the straight line segments from the real traffic image, some invalid line segments should be removed. This will lead to a fact that four collinear points are difficult to find in a road image. Although the longer the detected line segment indicates the more pixels in the same direction, the shorter ones will play an even important role in the process. To compute the reasonable cross-ratio value, we make an auxiliary line in term with the shortest line segment that owning the same direction with the longer ones. Then we can guarantee the existing of the C_r_ under the extreme worse environment. And then use the invariant cross-ratio to check the potential existence of pattern of pedestrian crossings. To decrease other false alarms efficiently, a variation of the cross-ratio constraint can be utilized to provide this geometrical configuration. For line groups, the required C_r_ can always be varied by permuting the four image points in order to obtain a more accurate detection result. This technique can be used to validate all potential candidate ROIs and be helpful for resulting in a lower false positive rate without causing loss of important information.

#### 3.2.2. Intensity Information Constraint

The color feature has received significant attention from researchers because of the large amount of information contained in images. Even though the information contained in a traffic image is very complex, color difference measurements will be of additional help in achieving coherent partitioning of the color space. Videos from probe vehicles involve varying road surface conditions and changing illumination; color information for patterns of pedestrian crossings thus becomes even more complicated because of these physical factors.

Compared with RGB color space, LST space is a better representation of luminance-chrominance characteristics [32]. The transformation C from RGB to LST is given by

(2) $$\begin{array}{cc} I_{\mathrm{LST}}\left(x,y\right)= & {\mathrm{CI}}_{\mathrm{RGB}}\left(x,y\right)=\left[\begin{array}{c} I_{L}\left(x,y\right)\\ I_{S}\left(x,y\right)\\ I_{T}\left(x,y\right) \end{array}\right]=\left[\begin{array}{ccc} \frac{\alpha }{\sqrt{3}} & \frac{\alpha }{\sqrt{3}} & \frac{\alpha }{\sqrt{3}}\\ \frac{\alpha }{\sqrt{2}} & 0 & -\frac{\alpha }{\sqrt{2}}\\ \frac{\alpha }{\sqrt{6}} & -2\frac{\alpha }{\sqrt{3}} & \frac{\alpha }{\sqrt{6}} \end{array}\right]\left[\begin{array}{c} I_{R}\left(x,y\right)\\ I_{G}\left(x,y\right)\\ I_{B}\left(x,y\right) \end{array}\right] \end{array}$$

where $\alpha =255/\max\{I_{R}\left(x,y\right),I_{G}\left(x,y\right),I_{B}\left(x,y\right)\}$. Note that the chrominance components ST in LST color space will not vary with intensity changes and S component can represent light variations correctly. This is an importance point, as it is well known that the spectral characteristic of natural and artificial illuminant can vary considerably [33]. Hence, an efficient strategy capable of adapting to illumination changes and extracting the candidate region should be considered.

We first represent the candidate ROIs using LST color space and then compute the mean intensity value. Let F(x,y) denote the candidate region of interest of size Width×Height, and let f(x,y) represent the intensity value of pixel (x,y). Let *m* and *d* be the mean and standard deviation, respectively, of the intensity values for a ROI with a size Width×Height. We can compute *m* and *d* by

(3) $$\begin{array}{c} m=\frac{\sum_{j=1}^{n}\sum_{i=1}^{p}f\left(x,y\right)}{Width\cdot Height}\\ d=\sqrt{\frac{\sum_{j=1}^{n}\sum_{i=1}^{p}{\left[f\left(i,j\right)-m\left(x,y\right)\right]}^{2}}{Width\cdot Height}} \end{array}$$

As the statistical analysis for the pedestrian crossings, a confidence interval can be calculated from the mean intensity value and standard deviation in a local ROI of size Width∗Height. Note that all positive and negative samples are from the pre-existing detection results randomly and the sample size of each set is 500. We use a statistical estimation method to derive a proper confidence interval for multiple pedestrian crossing detection. By experimentation, we can find that an appropriate threshold for pedestrian crossing detection is in the range of (75, 165).

#### 3.2.3. Aspect Ratio Constraint

**Figure 7:**
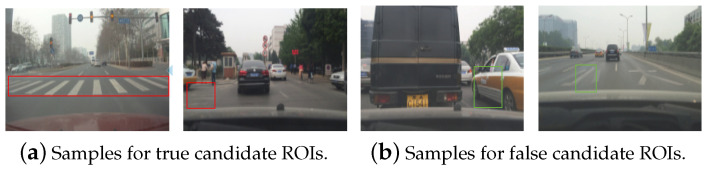
Samples for the true candidate ROIs and false candidate ROIs.

Because noises are inherent in real-world traffic settings, false alarms can occur because of variations in the viewpoint of the camera. Since the pedestrian crossings will always be essentially rectangular, they will become even flatter when the vehicles are nearer to the crossings. When taking into account the factor of distance, the varying aspect ratio for pedestrian crossings can fall in an appropriate scope. To estimate the normal scope of aspect ratio effectively, 600 preliminary detection results are used for statistical analysis. Figure 7a,b show the true and false candidate ROIs. Based on the statistical computing, the result reveals that the specific values for aspect ratio is at [1,8.65]. This scheme can improve the performance of method by filtering out the detection results from random noises.

## 4. Experimentation and Evaluation

In this section, we conduct experiments to evaluate the proposed approach using probe vehicle videos recorded in Beijing and Shanghai cities. Then, based on the training stage, we can obtain the key parameters for the proposed method. Then we investigate the generalization of the model for multiple pedestrian crossings in one traffic image on the validation set. Finally, we give the comparative experiments under the different conditions on the testing set.

### 4.1. Experimental Data

For pedestrian crossing detection, there are different experimental datasets due to different application scenarios and purposes [4,5,7]. These datasets are mainly built for the single pedestrian crossing detection. However, the main purpose of this work is to detect multiple pedestrian crossings in real traffic images oriented to real-time navigation, we collect the experimental data based on probe vehicles. To pursue an efficient multiple pedestrian crossing detection method, we use 10 videos from probe vehicles recorded in Beijing and Shanghai cities. These videos include situations of different partial occlusion by neighboring vehicles or pedestrians, deformations, lighting changes, and so on. Each of these videos lasts 15 minutes and is recorded at 29.97 frames per second. We first convert these videos to camera images by retaining every fifth frame. These images are classified into groups based on the numbers of pedestrian crossing. We then sample from these groups to form our final data set, which has a total of 1,379 images, of which 319 contain no pedestrian crossing, 441 contain one pedestrian crossing, 528 contain two pedestrian crossings, and 91 contain three pedestrian crossings. These images are split into three data sets according to the number of pedestrian crossing in image randomly, denoted as D1 (training set), D2 (validation set) and D3 (testing set):

- D1: Contains a total of 331 frames, of which 82 contain no pedestrian crossings, 101 contain one pedestrian crossing, 128 contain two pedestrian crossings, and 20 contain three pedestrian crossings. The total number of pedestrian crossing is 417; and
- D2: Contains a total of 468 frames, of which 97 contain no pedestrian crossings, 140 contain one pedestrian crossing, 200 contain two pedestrian crossings, and 31 contain three pedestrian crossings. The total number of pedestrian crossings is 633; and
- D3: Contains a total of 580 frames, of which 140 contain no pedestrian crossings, 200 contain one pedestrian crossing, 200 contain two pedestrian crossings, and 40 contain three pedestrian crossings. The total number of pedestrian crossings is 720.

Therefore, existing pedestrian crossing detection methods report the experimental results using two metrics: average recall ratio and average precision ratio which can measure the percentage of correctly detected objects on their own datasets. To comparison our method with different methods, we also use the same mean measurement. However, to give more impression of multiple pedestrian crossing detection, we also give more analyze about the whole performance of our method. For example, we will treat it as a failure even though one object is missing when an image contains three pedestrian crossings.

### 4.2. Training

#### 4.2.1. Vanishing Points Detection

During the training procedure on dataset D1, we obtain key parameter values for the presented algorithm. For vanishing point searching, we investigate parameter selection in the Hough space. We can empirical found that when the number of supporting lines is less than four for an intersection point, there will be no similar pattern of pedestrian crossing. In the 3D ordination system, any pedestrian crossings will always occur in the image plane. Then to guarantee the efficient and accurate of the algorithm, we can set the parameters θ and φ to π/4 and π/10 intervals respectively to accelerate the searching speed during quantization of the Hough space. Then we use the the value of ΔS to obtain an accumulator on the θ, φ plane to determine the potential vanishing points.

For data set D1, there are only five cases in which the vanishing point were completely missed. Among the experimental results, one case containing three pedestrian crossings are not detected because of the camera’s viewing angle, two cases containing two crosswalks were detected unsuccessfully, and two cases containing one pedestrian crossing are not fully detected because of serious staining on the crosswalk. We can see that the proposed method can detect existing vanishing points quickly under different road conditions; this will be of benefit for finding multiple pedestrian crossings efficiently.

#### 4.2.2. Concurrent Lines

Another area of concern is the number of concurrent lines when lines are grouped together as a region of interest for pedestrian crossing detection. To avoid a high number of false alarms, having a greater number of concurrent lines could serve to filter out those false targets. Hence, we investigate this strategy by conducting experiments to determine a practical parameter value for the number of concurrent lines selected on data set D1.

**Figure 8:**
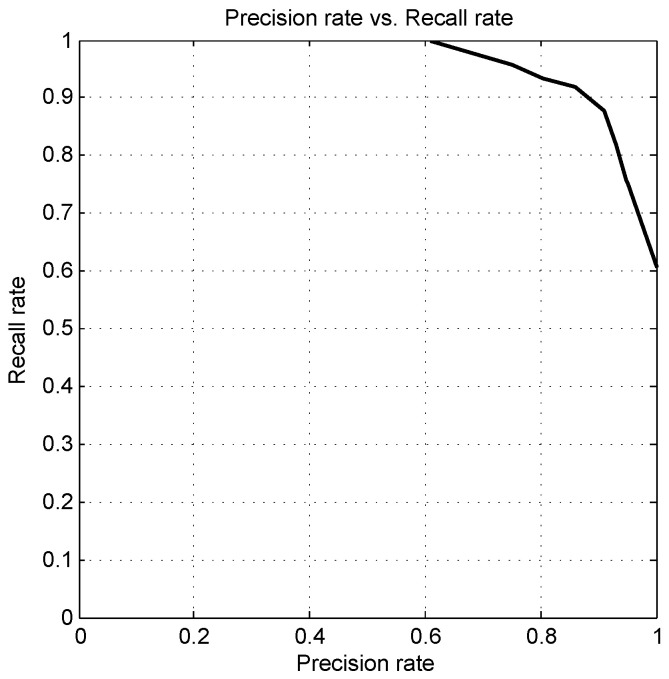
Detection precision-recall curve on D1.

We conduct a series experiments with the varying number of concurrent lines from four to ten. Empirically, we find that the precision rate improved significantly, from 60.5% to 100%, while the recall rate falling from 100% to 60.3%. When the number of concurrent lines is equal to six, the best trade-off between the recall rate and the precision rate is achieved: a recall rate of 91.1% and a precision rate of 86.8%. Therefore, we take six as a reasonable value for this parameter; this could take the form of three white bands or six segments to support the detection of a pedestrian crossing. Under the condition of vehicle-mounted, a three-stripe pattern can also form the basis of a pedestrian crossing finding. This can be used as a basic factor for mitigating the rate of false alarms, while preserving other candidate ROIs for further investigation. Figure 8 shows the curve of precision rate vs. recall rate.

### 4.3. Comparison at Different Steps

At the coarse stage, we aim to discover the potential ROIs to assure a high recall rate, more false alarms should be filtered out to guarantee the precision rate. To illustrate the effect of exploring prior constraints, we perform experiments to compare the effectiveness after each step of the fine stage on data set D1. The detection results are reported in Table 1. From this table, we can see that the false negative rate is sharply reduced when incorporating a priori information step by step, while the true positives are not lost significantly.

At the fine stage, the cross-ratio constraint information provides a strong geometric filter for refining the candidate ROIs generated by the coarse stage. The precision rate at this step increases by 4.16%, while the recall rate has not decreased markedly. The false alarms caused by redundant lines from neighboring targets are filtered out, mitigating the potential interference from markings for lanes, emergency stop areas, and so on. Under the constraint of intensity, the aggressive candidate ROIs have been discarded, improving the precision rate from 82.95% to 86.46% at a cost of losing 5 true pedestrian crossings. Further a priori information from the aspect ratio is useful for eliminating other false negatives caused by random objects, e.g., trees and buildings, obtaining the final recall rate 91.59% and precision rate 90.07% respectively. Thus, the proposed framework converges to provide satisfying detection results for multiple pedestrian crossings in camera images.

### 4.4. Validation

To conform the generalization of the proposed method, we conduct the cross validation experiments on D2 dataset. Since we want to identify the performance of the suggested method, we split the dataset D2 randomly according to the number of the pedestrian crossing. We first use all frames that contain zero and one pedestrian crossing as training samples to detect the instances which contain two objects and three objects respectively. Then, we use frames that contain zero, one and two pedestrian crossing as training samples to detect the instances which contain three objects.

**Figure 9:**
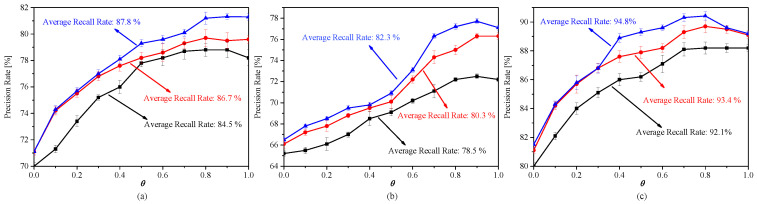
Validation results by using different kinds of samples to detect the multiple pedestrian crossing with different strategies of step-size. (**a**) The variable of precision ratio and recall ratio with the fixed-step π/4 by using zero and one samples to predict the detection of two pedestrian crossing. (**b**) The variable of precision ratio and recall ratio with the fixed-step π/10 by using zero and one samples to predict the detection of three pedestrian crossing. (**c**) The variable of precision ratio and recall ratio with the adaptive variable-step by using zero, one and two samples to predict the detection of three pedestrian crossing. Note that the blue, red and black lines are denoted the adaptive variable-step, fixed-step π/4 and fixed-step π/10 respectively.

To detect pedestrian crossing efficiently, we use the different strategies for the maximum step size θ during the method: fixed-step π/4, fixed-step π/10 and an adaptive variable-step for the proposed framework. The results of our validation experiments are shown in Figure 9. We can see that there are significant difference when applied different strategy of step size. Note that in the Figure 9a–c, the lines represent the variable of precision ratio according to the variable of main parameter θ during the vanishing point searching procedure: the blue curve denote the performance of adaptive variable-step, and the red and the black lines for the fixed-step π/4 and π/10 respectively. It is clear that when increasing levels of parameter θ from 0 to 1 for the quantizition of Hough space, the precision rate is increasing seriously. But when we use the adaptive variable-step, the precision rate and the recall rate are better than the other fixed-step schemes. The main reason is much more noises in the traffic images. When we use a smaller fixed-step, the disturbance will be more significantly for the framework. And when we use an adaptive scheme from 0 to 1 according to the experimental results, an even better trade-off of the precision ratio and recall ratio can be obtained. Another truth can be confirmed that a smart searching strategy will be better for an unseen traffic image while detect pedestrian crossing.

### 4.5. Comprehensive Assessment

#### 4.5.1. Multiple Pedestrian Crossing Detection

Road markings and characteristics can vary greatly not only between regions, but also between nearby stretches of road. Our objective is to carry out a comprehensive assessment of the proposed method under variations in illumination that are expected to be encountered in normal traffic flow. The overall experimental results of multiple pedestrian crossing detection on dataset D3 are summarized in Table 2.

From the Table 2, we can see that the proposed method is quite successful for detecting multiple pedestrian crossings in different traffic scenes. There are only 38 cases out of 720 pedestrian crossings that are not successfully detected. Among these, in 12 cases less than four white bands are contained within the camera’s viewing angle, and in 26 cases there are occlusion by neighboring vehicles. Note that for the 40 images containing three crossings, in 4 cases two crossings are missed. For the 200 images containing two crossings, in 3 cases both crossings are missed, and in 20 cases one crossing is missed. For the 200 images containing only one crossing, the detection framework missed only 6 cases. Overall, the experiment using the D3 data set gave a recall rate of 94.72% and a precision rate of 90.69%.

**Figure 10:**
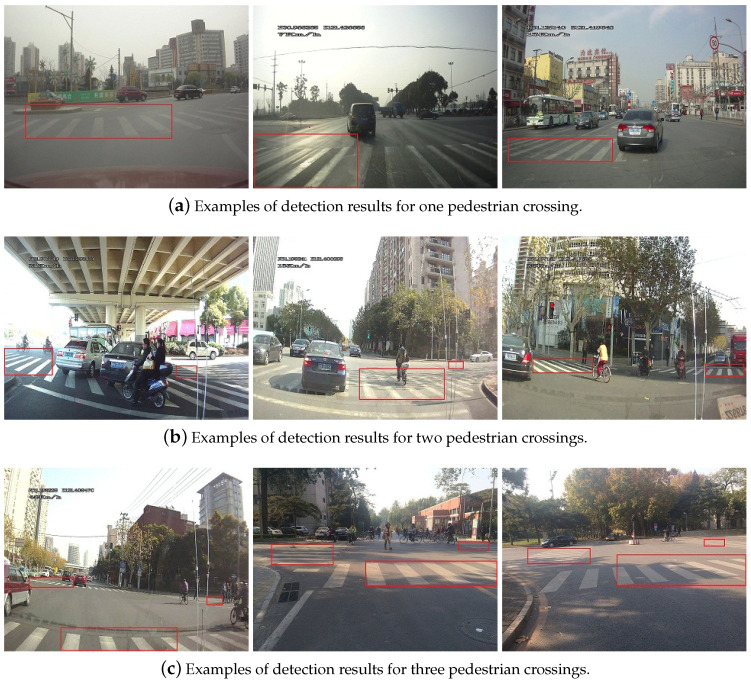
Examples of detection results with varying number of pedestrian crossings in the camera image.

**Figure 11:**
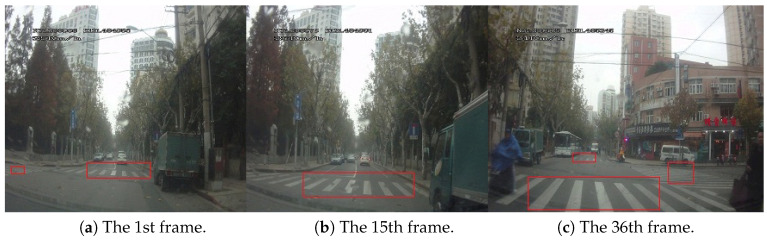
Frames from video showing detection of multiple pedestrian crossings in real traffic scenes.

Some examples of multiple pedestrian crossings detection results on D3 are showed in Figure 10, and Figure 11. Experimental analysis find that when the visibility of pedestrian crossing was worse, e.g., when the roadways and intersections are higher traffic volume or under special weather conditions, the results for multiple pedestrian crossing detection be worse, unsurprisingly. Meanwhile, when the visual feature of pedestrian crossing are indistinct, the number of random false targets increased seriously, indicating the necessity of the prior constraint information for multiple pedestrian crossing detection.

#### 4.5.2. Comparison with Prior Methods

The prior methods focus on detecting one pedestrian crossing which immediately appear in front of the camera so they can not detect more than one pedestrian crossings in one image. In this comparative experiment, the testing data is from the dataset D3, containing zero or one pedestrian crossing. The total number of frames is 340, of which 140 contain no pedestrian crossings, 200 contain one pedestrian crossing. The complete experimental results for different methods are summarized in Table 3.

As shown in Table 3, the proposed method has a higher recall rate 96.85%, while preserves a higher precision rate 95.4%. Under the varying condition of illumination or orientation changes in camera images, unstable visual information and geometric transform can lead to imperfect experiment result for [4,5,12]. Meanwhile, the grouping geometric features in figure-ground segmentation tend to be fail when pedestrian crossing is occluded partially [6,14]. On the one hand, experimental analysis show that we can also obtain an even better pedestrian crossing detection result under the same experiment condition comparing to other classical methods.The proposed framework has an advantage to detect the pedestrian crossing by integrating visual cues and priori constraint information, since it can search all potential candidate ROIs in camera images at the coarse stage. On the other hand, since the essential characteristic is specially designed for pedestrian crossing, it is reasonable to explore some prior constraint information to refine and identify the true ROIs, and that it involves optimized post-processing steps.

## 5. Discussion and Future Work

In this paper, we established an efficient framework for task-driven multiple pedestrian crossing detection. Besides, we provide the experimental results based on our datasets.

Unlike most existing methods, which only provide a single pedestrian crossing detection, our task-driven method also provide a framework of exploring all pedestrian crossing detection which captured by a camera. According to the obtained vanishing points, visual features and constraints cues, we have some interesting observations. Given the same image which contains no pedestrian crossing, although there are some noises, our framework can be very efficient. The proposed method tend to focus on whole regions of interest, but mainly focus on the vanishing points detection at the right directions in the image. When there is only one true pedestrian crossing in an image, we will summary much similar results with [4,6]. Experimental results reveal that the methods tend to fixate prior on clear objects in an image. The above phenomenon confirm that both vanishing points detection and visual features are more significant factors influencing pedestrian crossing detection. However, our experimental results for multiple pedestrian crossing detection also illustrate the importance of prior constraints. Thus, we hope to raise critical thinking about more efficient methods for multiple pedestrian crossing detection in real traffic images.

Future work may involve how to improve the accuracy of the multiple pedestrian crossing detection under complex traffic scenes based on deep learning and designing evaluation protocol. We will consider the implementation of the time variable as a useful clue to improve the performance of our framework. Because the frames are very similar to the previous ones when the car moving, we can also improve the performance of the method by considering whether a crosswalk or multiple ones were already detected successfully in previous frames. We will further test our framework on bigger datasets which coming from probe vehicles under more complex traffic images to confirm its generalization and robust.

## Figures and Tables

**Table 1:** Summary of pedestrian crossing detection results at different steps on D1.

Scheme	Decision	Crossing	No crossing	Recall Rate (%)	Precision Rate (%)
Coarse stage	Crossing	405	12 (False positive)	97.12	78.79
	No crossing	109 (False negative)	-		
Cross-ratio	Crossing	394	28 (False positive)	94.48	82.95
	No crossing	81 (False negative)	-		
Intensity cue	Crossing	383	33 (False positive)	92.07	86.46
	No crossing	60 (False negative)	-		
Aspect-ratio	Crossing	381	35 (False positive)	91.59	90.07
	No crossing	42 (False negative)	-		

**Table 2:** Summary of pedestrian crossing detection results on data set D3.

Type (♯ Frames)	Decision	Crossing	No Crossing	Recall Rate (%)	Precision Rate (%)
0 (140)	Cross	-	-	-	-
	No crossing	11 (False negative)	-		
1 (200)	Cross	194	6 (False positive)	97	95.10
	No crossing	10 (False negative)	-		
2 (200)	Cross	376	24 (False positive)	94	91.04
	No crossing	37 (False negative)	-		
3 (40)	Crossing	112	8 (False positive)	93.33	90.32
	No crossing	12 (False negative)	-		
Total (580)	Cross	682	38 (False positive)	94.72	90.69
	No crossing	70 (False negative)	-		

**Table 3:** Summary of pedestrian crossing detection results using different methods.

Scheme	Decision	Crossing	No Crossing	Recall Rate (%)	Precision Rate (%)
[5]	Cross	158	42 (False positive)	71.9	70.22
	No crossing	67 (False negative)	-		
[12]	Cross	163	37 (False positive)	81.5	76.17
	No crossing	51 (False negative)	-		
[14]	Cross	187	13 (False positive)	93.5	87.38
	No crossing	27 (False negative)	-		
[4]	Cross	189	11 (False positive)	94.5	90.87
	No crossing	19 (False negative)	-		
[6]	Cross	191	9 (False positive)	95.5	91.39
	No crossing	18 (False negative)	-		
Our method	Cross	194	6 (False positive)	97	95.1
	No crossing	10 (False negative)	-		
